# Aberrant extrathymic T cell receptor gene rearrangement in the small intestinal mucosa: a risk factor for coeliac disease?

**DOI:** 10.1136/gut.2007.125526

**Published:** 2008-02-25

**Authors:** A Bas, G Forsberg, V Sjöberg, S Hammarström, O Hernell, M-L Hammarström

**Affiliations:** 1 Department of Clinical Microbiology, Immunology, Umeå University, Umeå, Sweden; 2 Department of Clinical Sciences, Pediatrics, Umeå University, Umeå, Sweden

## Abstract

**Background:**

Coeliac disease is a small intestine enteropathy caused by permanent intolerance to wheat gluten. Gluten intake by patients with coeliac disease provokes a strong reaction by intestinal intraepithelial lymphocytes (IELs), which normalises on a gluten-free diet.

**Aim:**

To investigate whether impaired extrathymic T cell maturation and/or secondary T cell receptor (TCR) gene recombination in IELs are features of coeliac disease which could contribute to the failure of establishing tolerance to gluten.

**Methods:**

Expression levels of the four splice-forms of recombination activating gene-1 (RAG1) mRNA and preT α-chain (preTα) mRNA were determined in IEL-subsets of children with coeliac disease and controls. Frequencies of RAG1 expressing IELs were determined by immunomorphometry.

**Results:**

In controls, the RAG1-1A/2 splice-form selectively expressed outside the thymus, was dominant and expressed in both mature (TCR^+^) and immature (CD2^+^CD7^+^TCR^−^) IELs (∼8 mRNA copies/18S rRNA U). PreTα was expressed almost exclusively in CD2^+^CD7^+^TCR^−^ IELs (∼40 mRNA copies/18S rRNA U). By contrast, RAG1 and preTα mRNA levels were low in patients with coeliac disease compared to controls, both with active disease and with inactive, symptom-free disease on a gluten-free diet (p values <0.01 for mature and <0.05 for immature IELs). Similarly, the frequencies of RAG1+ IELs were significantly lower in patients with coeliac disease compared to controls (p<0.001).

**Conclusions:**

Patients with coeliac disease appear to have an impaired capacity for extrathymic TCR gene rearrangement. This is an inherent feature, which probably plays a pivotal role in the failure to efficiently downregulate the T cell response to gluten.

Coeliac disease (CD) is a chronic inflammatory disease of the small intestine affecting genetically susceptible individuals carrying the HLA-DQ2 and/or HLA-DQ8 alleles. It is caused by failure to establish and/or maintain tolerance to dietary prolamins in wheat, barley and rye, and particularly to wheat gliadin.^1^ Active disease is associated with an intestinal lesion, typically showing villous atrophy, crypt hyperplasia, and increased numbers of lymphocytes within both the epithelium and the lamina propria. Clinical and histological improvements are seen upon withdrawal of wheat, barley and rye from the diet.^2^ A central role for T lymphocytes in coeliac disease has long been recognised,^3^ and gliadin-specific T cell clones have been isolated from the small intestinal mucosa of patients with coeliac disease.^4^ ^5^

Accumulating evidence suggests an important role for intraepithelial lymphocytes (IELs) in coeliac disease pathogenesis. Human small intestinal IELs are composed of multiple T lymphocyte subsets; the major subsets being CD4^+^ and CD8^+^ αβ T cells, CD4CD8 double-negative γδ T cells, and cells with a thymocyte-like phenotype (CD1a^+^ cells, CD2^+^CD3^−^ cells and CD4^+^CD8^+^ cells).^6^ ^7^ The numbers of both αβIELs and γδIELs are increased in the small intestine of patients with coeliac disease, and although the number of αβIELs varies with disease activity, it is uncertain whether this also is the case for γδIELs, which seem to stay elevated longer after gluten has been withdrawn from the diet.^8^^–^10 In addition, the otherwise rare CD8^+^ IEL subtype expressing CD94, ie, one of the two chains in several natural killer (NK)-cell receptors, is increased in active coeliac disease.^11^ Moreover, the increased production of interferon γ by T cells in active coeliac disease is most pronounced in IELs,^12^ and IELs from patients with coeliac disease exhibit cytotoxicity regulated in an NK-cell-like manner.^13^^–^15

IELs in non-coeliac adults express recombination activating gene-1 (RAG1) and pre T α-chain (preTα) mRNAs.^16^ Up to 6% of IELs expressed the RAG1 protein, a finding that suggests ongoing T cell receptor (TCR) gene rearrangement. The human RAG1 gene has three different 5′ untranslated region (UTR) exons that generate four different mRNA splice forms, two of which are expressed exclusively outside the thymus.^16^

We hypothesised that abrogated or decreased TCR recombination, either in de novo extrathymic T cell maturation or in TCR editing in mature T cells, may negatively influence establishment and/or maintenance of tolerance to food antigens. Therefore, we compared expression levels of RAG1 mRNA splice forms and preTα mRNA in T cell lineage subsets of IELs, using biopsies from children with coeliac disease, both in active and inactive disease, and from children with no food intolerance. The frequencies of IELs expressing the RAG1 protein were also compared between patients with coeliac disease and controls. Coeliac disease is an excellent human model for studies of this type since the nominal antigen is known and inflammation ceases upon its withdrawal from the diet. Thus, the availability of intestinal biopsies from patients with active and inactive disease allows discrimination between genetically determined properties and inflammation-dependent properties.

## METHODS

### Patients and biopsy sampling

Intestinal biopsies were collected from children admitted to the Department of Pediatrics at Umeå University Hospital on suspicion of coeliac disease or for evaluation of asymptomatic growth failure or short stature. Small intestinal biopsies were collected from distal duodenum/proximal jejunum at the level of the ligament of Treitz, using a Watson paediatric capsule. Part of the biopsy sample was used for routine pathology examination and grading by the Alexander score and the rest for isolation of IELs or for immunohistochemistry. Biopsies were immediately placed in ice-chilled HEPES-buffered RPMI 1640. Patients were from three diagnostic groups, as follows.

- *Active coeliac disease*: children showing increased anti-endomysium immunoglobulin A (IgA) (AEA) and/or anti-gliadin IgA (AGA) antibody titres and a jejunal mucosa with increased numbers of IELs and with villous atrophy and crypt hyperplasia; all responding positively to a gluten-free diet. (Nineteen children: six boys and 13 girls, median age 4.3 years (range, 1.3–17.0 years); AEA titres, 40 to ⩾320; Alexander scores, 2 (one child) and 3–4.)
- *Treated coeliac disease*: children with coeliac disease who had been on a gluten-free diet for ⩾11 months. (Thirteen children: eight boys and five girls, median age 10.2 years (range, 4.5–19.0 years); AEA titres, ⩽20; Alexander score, 1.)
- *Controls*: children with no known food intolerance. (Twenty children: 11 boys and nine girls, median age 3.5 years (range, 1.1–18.2 years); AEA titres, ⩽20, Alexander score, 1.)

Oral informed consent was obtained from the patient and/or the parents.

### Cell isolation procedures

**Figure 1:**
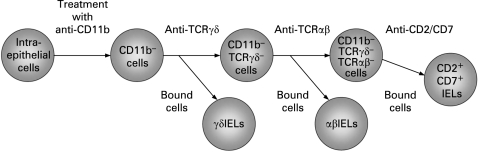
Isolation procedure for intraepithelial lymphocyte (IEL) subtypes from intestinal biopsies. Rinsed tissue was treated with dithiothreitol and vigorous shaking, and free intraepithelial cells were subjected to the selection procedure. First, cells of myeloid lineage and inherently sticky cells were removed by treatment with anti-CD11b monoclonal antibody (mAb)-coated paramagnetic beads. Unbound cells (CD11b^−^ cells) were collected and subjected to sequential positive selection by incubation first with paramagnetic beads coated with a mixture of anti-TCRγδ mAbs (γδIELs), followed by incubation of unbound cells with paramagnetic beads coated with anti-TCRαβ mAb (αβIELs), and finally incubation of unbound cells with a mixture of anti-CD2 mAb and anti-CD7 mAb coated paramagnetic beads (CD2^+^CD7^+^IELs). RNA was subsequently extracted from the bound cell fractions.

Intestinal IELs were isolated from small intestinal biopsies as previously described.^12^ The procedure includes pretreatment with anti-CD11b mAb (clone OKM1; ATCC, Rockville, Maryland, USA) charged paramagnetic beads (Dynabeads M-450 coated with goat-anti-mouse IgG; Dynal, Oslo, Norway). Subpopulations of IELs were thereafter retrieved by sequential positive selection as depicted in fig 1, using paramagnetic beads charged with anti-TCRγδ mAbs (anti-Vδ1 clone TS-8.2, anti-TCRδ-chain clone 5A6.E9 plus anti-TCRγ-chain clone Y3.30; all from Serotec, Kidlington, Oxford, UK), anti-TCRαβ mAb (clone BMA031; Serotec), and finally, a mixture of beads charged with anti-CD7 mAb (clone DK24; Dakopatts, Glostrup, Denmark) and anti-CD2 directly coated on the beads (Dynal). Microscopic inspection of all positively selected samples was performed to ascertain that only cells with surface-bound beads were present. The small amounts of cells in IEL subpopulations retrieved from single biopsies precluded parallel RNA and phenotypical analyses. However, previous studies have shown that fewer than 4.6% of the respective surface marker-positive cells can be found in the unbound fraction and that >98% of the marker-positive cells were bound to the relevant beads using this procedure.^7^ ^12^ ^16^ The isolation procedure was started within 45 min following biopsy and performed at 4°C, and positively selected cells were frozen within 1 h after exposure to mAb.

### RNA preparation

Total RNA was extracted from the different cell preparations by the acid guanidinium thiocyanate/phenol/chloroform method and dissolved in RNase-free water containing rRNAsin ribonuclease inhibitor (Promega, Madison, Wisconsin, USA), as described.^7^ ^12^

### Real-time quantitative RT-PCR

Quantification of the 1A/2, 1B/2, 1A/1B/2, and 1C/2 splice forms of RAG1 mRNA and the long isoform of preTα, preTα^a^ was performed using real-time quantitative RT-PCR assays with RNA copy standards constructed in the laboratory.^16^ Samples were analysed in triplicate and expressed as copies of mRNA per microlitre. The concentration of 18S rRNA was determined in each sample using real-time quantitative RT-PCR (Applied Biosystems, Foster City, California, USA), and the results were expressed as mRNA copies per 18S rRNA U. One 18S rRNA U was defined as the signal obtained by 10 pg of a pool of total RNA extracted from peripheral blood mononuclear cells (PBMCs) stimulated with anti-CD3 mAb, which corresponds to approximately 100 lymphocytes.^17^ All samples included in the study contained >19.5 U 18S rRNA per reaction mixture.

### Cloning and sequencing

After gel electrophoresis, RT-PCR products for preTα^a^ mRNA were isolated, ligated into *Eco*RV-digested pBluescript SK II(+) prepared with a dT overhang, transformed into competent *Escherichia coli* XL-1 Blue, cloned, and sequenced as previously described.^16^

### Immunohistochemistry

Fresh tissue samples were snap frozen in liquid nitrogen and stored at −80°C. Cryostat sections (8 μm thick) were air-dried, fixed in 4% paraformaldehyde for 15 min at room temperature and then rinsed in cold 0.02 mol/l phosphate-buffered saline (PBS, pH 7.4). Free aldehyde groups were blocked by incubation with 0.1 mol/l glycine in PBS and sticky nuclear sites were blocked by incubation with 10% acetic acid. Sections were further blocked and membranes permeabilised by incubation with PBS containing 0.2% bovine serum albumin/0.05% saponin/0.1% Triton-X100 followed by incubation with 2.5% normal horse serum (Vector Laboratories, Burlingame, California, USA). Thereafter the sections were incubated for 1 h with the IgG fraction of rabbit anti-human RAG1 antiserum (sc-5599; Santa Cruz Biotechnology, Santa Cruz, California, USA) diluted in PBS containing 0.2% bovine serum albumin/0.05% saponin/0.1% Triton-X100. Endogenous peroxidase activity was blocked by incubation with 0.03% H_2_O_2_ and 13 mmol/l NaN_3_ at 37°C for 45 min. The slides were washed and incubated for 60 min with ImmPress anti-rabbit Ig reagent (Vector Laboratories). Positive cells were visualised by incubation with diaminobenzidine substrate and 0.03% H_2_O_2_ in 0.05 mol/l Tris–HCl buffer (pH 7.6). Finally, sections were counterstained with methyl green and mounted in Canada balsam. Sections incubated with concentration-matched IgG fraction of normal rabbit serum (Dakopatts) served as negative controls and sections incubated with the IgG fraction of rabbit anti-human CD3 antiserum (Dakopatts) served as positive controls. Morphometry was performed by inspecting the entire section for stained IELs and measuring the total epithelial length in the section by using an integrating, cooled colour 3CCD camera (Colour Chilled 3 CCD Hamamatsu Camera C5810; Hamamatsu Photonics, Hamamatsu City, Japan) on a standard light microscope combined with an interactive computer image analysis system (LeicaQWin; Leica Imaging Systems, Cambridge, UK).

### Statistics

Statistical analysis was performed using the Prism 4 computer program (GraphPad Software, San Diego, California, USA). Samples within groups were tested for Gaussian distribution by the Kolmogorov–Smirnov normality test. Statistical analysis of differences in RAG1 and preTα mRNA expression levels between IEL subtypes and frequencies of RAG1^+^ IELs in the three patient groups was performed using one-way analysis of variance (ANOVA) with the Bonferroni multiple comparison post-test. A p value <0.05 was regarded as statistically significant.

## RESULTS

### T cell maturation and TCR editing normally occur in parallel

The expression patterns of RAG1 and preTα mRNAs in IELs suggest that extrathymic T cell maturation and TCR editing/revision normally take place in parallel in the small intestinal epithelium of children. IELs were isolated from small intestinal biopsies and directly subjected to sequential positive selection, as depicted in fig 1 and levels of the 1A/2, 1A/1B/2, 1B/2, and 1C/2 splice forms of RAG1 mRNA and the long isoform of preTα were determined in the γδIELs, αβIELs, and CD2^+^CD7^+^IELs.

**Figure 2:**
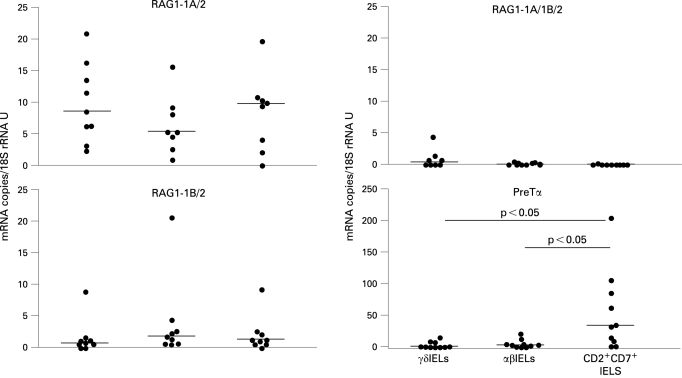
Recombination activating gene 1A/2 (RAG1-1A/2) mRNA is the splice form preferentially expressed in small intestinal intraepithelial lymphocytes (IELs) of children and is expressed at similar levels in both mature and immature IELs, while preTα mRNA is expressed at high levels only in immature IELs. Expression levels of the 1A/2, 1B/2, and 1A/1B/2 RAG1 mRNA splice forms and preTα mRNA in γδIELs, αβIELs, and CD2^+^CD7^+^IELs retrieved from small intestinal biopsies of controls. p Values of statistically significant differences are depicted. Dots indicate expression levels for the indicated mRNA species in individual IEL subtype samples. Horizontal bars indicate median values.

RAG1 mRNA was detected in all control IEL samples and in all three subsets thereof. The RAG1-1A/2 splice form, which is exclusively expressed outside the thymus, was expressed in the highest concentrations, followed by the RAG1-1B/2 splice form (fig 2). The long 1A/1B/2 splice form of RAG1 was detected only in occasional samples (fig 2). There was no significant difference in expression levels between the IEL subtypes. In accordance with previous results,^16^ the thymus-selective RAG1-1C/2 splice form was not detected in any of the IEL subtypes (n = 3; data not shown).

As expected, preTα mRNA was mainly expressed in immature CD2^+^CD7^+^IELs (median 36 copies per 18S rRNA U compared to ∼1 copy per 18S rRNA U in IELs expressing TCR; fig 2). Cloning and sequencing of the RT-PCR products confirmed the expected sequence of preTα mRNA.

**Figure 3:**
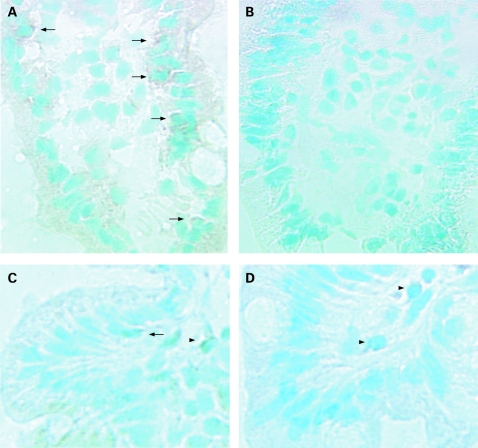
Lymphocytes expressing the recombination activating gene-1 (RAG1) protein are normally present within the small intestinal epithelium of children. Immunoperoxidase staining of small intestinal biopsies of two control patients with anti-RAG1 rabbit immunoglobulin G (IgG) (A,C,D). Cells with typical granular staining of the nucleus can be seen in villous epithelium (A), both villous epithelium and lamina propria (C), and in the lamina propria only (D). (B) Section from the same patient as in (A) incubated with IgG from normal rabbit serum. Arrows indicate intraepithelial lymphocytes (IELs) stained positively for RAG1, and arrowheads indicate RAG1^+^ cells in lamina propria. Original magnification, ×220.

Immunohistochemistry with anti-RAG1 antibodies revealed presence of RAG1 positive IELs (table 1 and fig 3). The RAG1^+^ cells exhibited a granular staining of the nucleus and showed an irregular distribution in the tissue. Occasional villi harboured small clusters of positive cells (fig 3A), while others had scattered positive IELs alone (fig 3C) or in the vicinity of RAG1^+^ lamina propria lymphocytes (fig 3D) and yet other villi were devoid of RAG1^+^ cells (data not shown).

**Table 1:** Frequencies of recombination activating gene 1 (RAG1) expressing intraepithelial lymphocytes (IELs) in the small intestinal mucosa of patients with coeliac disease and in controls

Patient group	Frequency of RAG1^+^ IELs*	n	p Value
Controls	0.33 (0.11)	5	
Active coeliac disease	0.05 (0.02)	6	<0.001
Treated coeliac disease	0.06 (0.02)	5	<0.001

n, number of samples counted.

p Values indicate statistically significant differences between patients with coeliac disease and controls.

*Mean (1 SD) of number of RAG1^+^ cells/mm epithelium.

### RAG1 expression is significantly decreased in IELs of patients with coeliac disease

The expression levels of the four RAG1 mRNA splice forms were also determined in γδIELs, αβIELs, and CD2^+^CD7^+^IELs isolated from intestinal biopsies of children with active, newly diagnosed untreated coeliac disease (active coeliac disease) and from symptom-free patients with coeliac disease on a gluten-free diet (treated coeliac disease).

**Figure 4:**
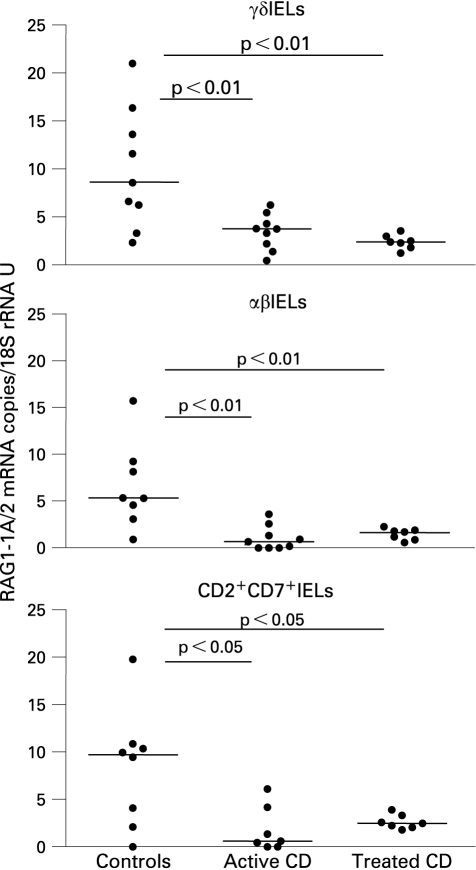
Expression levels of the recombination activating gene-1 (RAG1)-1A/2 mRNA splice form are significantly decreased in patients with coeliac disease. Expression levels of RAG1-1A/2 mRNA in γδIELs, αβIELs, and CD2^+^CD7^+^IELs freshly isolated from small intestinal biopsies of controls and patients with coeliac disease with active disease (active coeliac disease (Active CD)) and inactive disease (treated coeliac disease (Treated CD)). p Values of statistically significant differences are depicted. Dots indicate expression levels for the indicated mRNA species in individual IEL subtype samples. Horizontal bars indicate median values. IELs, intraepithelial lymphocytes.

The expression levels of the RAG1-1A/2 mRNA splice form were significantly reduced compared to controls in all three IEL subsets of patients with coeliac disease both with active and inactive disease (fig 4). For the two weakly expressed splice forms, 1B/2 and 1A/1B/2, we found no significant difference between patients with coeliac disease and controls in any of the three IEL subsets (n = 7–9 for each IEL subset in patients with active and treated coeliac disease, respectively; data not shown). The RAG1-1C/2 mRNA splice form was not detected in γδIELs, αβIELs, or CD2^+^CD7^+^IELs of patients with coeliac disease neither with active (n = 3) nor inactive disease (n = 3; data not shown).

In accordance with the low expression levels of RAG1 mRNA, we found very few RAG1^+^ IELs in the small intestine of children with coeliac disease irrespective of disease activity (table 1). The difference between patients with coeliac disease and controls was statistically highly significant (table 1).

### PreTα mRNA expression levels are also decreased in IELs of patients with coeliac disease

**Figure 5:**
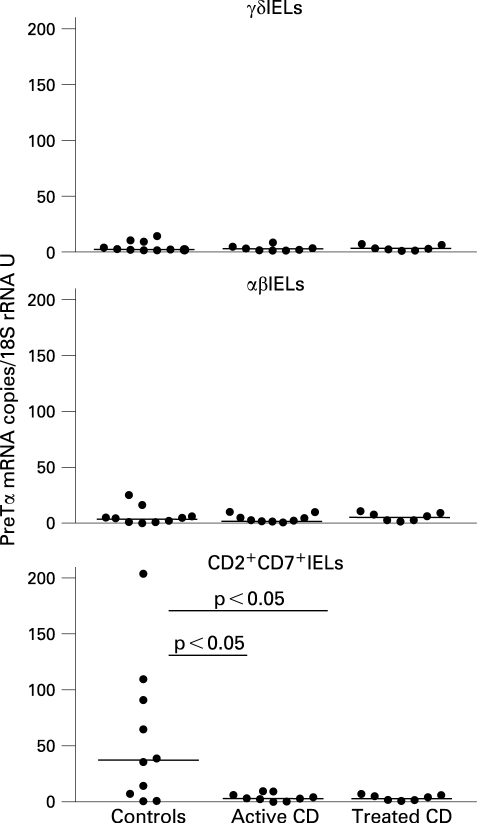
PreTα mRNA expression levels are significantly decreased in patients with coeliac disease. Expression levels of preTα mRNA in γδIELs, αβIELs, and CD2^+^CD7^+^IELs freshly isolated from small intestinal biopsies of controls and patients with coeliac disease with active disease (active coeliac disease (Active CD)) and inactive disease (treated coeliac disease (Treated CD)). p Values of statistically significant differences are depicted. Dots indicate expression levels for the indicated mRNA species in individual IEL subtype samples. Horizontal bars indicate median values. IELs, intraepithelial lymphocytes.

γδIELs, αβIELs, and CD2^+^CD7^+^IELs isolated from intestinal biopsies of children with active coeliac disease and treated coeliac disease were also analysed for expression levels of preTα mRNA. The preTα mRNA expression levels were significantly lower in the CD2^+^CD7^+^IELs of patients with coeliac disease, both in active and inactive disease, as compared to controls (fig 5). The expression levels of preTα mRNA in γδIELs and αβIELs were low with no difference between any of the coeliac disease groups and controls (fig 5).

## DISCUSSION

The results support three major conclusions. First, simultaneous expression of mRNAs for RAG1 and preTα, two proteins transiently expressed during T cell maturation, in IELs with the phenotype of immature T cell lineage cells (CD2^+^CD7^+^TCR^−^) in controls suggests that extrathymic T cell maturation normally occurs in the small intestinal epithelium of children. Taken together with previous findings,^6^ ^16^ ^18^ ^19^ these results suggest that extrathymic T cell maturation is a normal event in human small intestinal mucosa throughout life.

Second, we found that the IELs with a more mature phenotype, ie, αβIELs and γδIELs also expressed RAG1 mRNA suggesting ongoing TCR-gene rearrangement. The relatively high levels of RAG1 mRNA in γδIELs and αβIELs of children in the control group strongly suggest an important role for TCR revision by a secondary recombination of TCR gene segments in T cells located within the intestinal epithelium early in life. Most likely this reflects a T cell repertoire adaptation to the particular small intestinal milieu with its complex demands of effective protection without over-reaction to beneficiary components, eg, food antigens.

Third, children with coeliac disease have significantly decreased expression of both RAG1 in all three IEL-subtypes and of preTα mRNA in immature T cells, suggesting reduced extrathymic T cell maturation accompanied by decreased TCR editing and/or revision. This reduction was independent of disease activity, indicating that decreased T cell maturation in the intestinal mucosa is an inherent property of patients with coeliac disease. Thus, failure to adequately adapt the T cell repertoire to the milieu at the intestinal mucosal surface and its exposure to gluten peptides and the microbial flora might be an important factor in the pathogenesis of coeliac disease.

These results together with previous findings suggest that the small intestinal epithelium in patients with coeliac disease is inherently different in comparison to the epithelium of control individuals. As measured by lectin binding, patients with active coeliac disease show a different glycosylation pattern on the epithelial cells compared to controls and commonly have rod-shaped bacteria adhered to the epithelial surface.^20^ Patients with coeliac disease also seem to exhibit a different intestinal bacterial flora from controls, which persists on a gluten-free diet.^21^ Moreover, interferon γ levels decrease in IELs of patients with coeliac disease on a gluten-free diet; however, they seldom reach the low levels in IELs of controls.^12^ Furthermore, even though γδIELs is a minor cell population among IELs, it remains elevated for more than 1 year on a gluten-free diet.^10^ These results are compatible with the hypothesis that patients with coeliac disease have insufficient defence at the epithelial surface with poor discriminating capacity between pathogens and beneficial components, possibly because of interdependent differentiation of IELs and epithelial cell functions. A consequence could be that gluten peptides are mistaken for pathogens. The strong epithelial reaction with excessive interferon γ production by IELs and the presence of over-stimulated intraepithelial cytotoxic T lymphocytes, which have lost TCR restriction, are compatible with this idea.^12^ ^15^

Studies in mice^22^^–^26 have shown that there are two types of T cells maturing in the gut mucosa. These are precursor cells that develop into γδ T cells in the small intestine without thymic influence and immature thymus emigrants that colonise the small intestinal mucosa as T-cell-committed precursors and mature into IELs locally. Thus, it is an open question whether the immature IELs (CD2^+^CD7^+^TCR^−^ cells) seen expressing RAG1 and preTα mRNAs in the intestinal mucosa of children are early emigrants from thymus or are thymus-independent precursors of T cell lineage, or a mixture of both.

The significant reduction of the “extrathymic” RAG1-1A/2 splice form in both mature and immature T cell subsets and in both active and inactive disease, suggests that the tissue-specific signals required for involvement of the 1A exon in transcription of the RAG1 gene are low or lacking in patients with coeliac disease. The fact that both reduced RAG1 mRNA expression and decreased frequencies of IELs expressing the RAG1 protein is seen in both active disease and in inactive disease with normalised mucosal histology argues against “dilution” of cells undergoing TCR gene rearrangement with mature T cells recruited in the gluten-induced intestinal reaction. Instead, it underscores the likelihood that poor TCR gene rearrangement in the intestinal mucosa is an inherent feature of patients with coeliac disease. In line with our findings, Carton *et al*^27^ reported that the small CD4CD8 double positive IEL population, most likely the immature IELs, is significantly decreased in patients with coeliac disease, both in active and inactive disease. Together these findings strongly support the notion that defects in both extrathymic T cell maturation and TCR revision in mature T cells, limit the possibilities of escaping unwanted specificities against gluten epitopes and establishing tolerance in patients with coeliac disease.

It has been reported that interleukin 2 (IL)2-deficient mice have an impaired T cell lymphopoiesis in the gut and that the intestinal inflammation seen in these mice was caused by thymus-derived T cells.^28^ It is possible that the local T cell maturation also generates regulatory intestinal T cells in humans, in which case the reduced extrathymic T cell maturation in patients with coeliac disease would cause a reduced capacity to downregulate gluten-reactive, thymus-derived T cells in the intestinal mucosa.

In summary, it appears that reduced extrathymic T cell maturation and TCR fine-tuning are contributing factors to the inability to establish and maintain tolerance to gluten in patients with coeliac disease.
